# A tale of two parasites: Responses of honey bees infected with *Nosema ceranae* and *Lotmaria passim*

**DOI:** 10.1038/s41598-023-49189-9

**Published:** 2023-12-18

**Authors:** Courtney I. MacInnis, Lien T. Luong, Stephen F. Pernal

**Affiliations:** 1 Department of Biological Sciences, University of Alberta, Edmonton, AB T6G 2E9 Canada; 2 Agriculture and Agri-Food Canada, Beaverlodge Research Farm, P.O. Box 29, Beaverlodge, AB T0H 0C0 Canada

**Keywords:** Entomology, Parasite host response, Behavioural ecology

## Abstract

*Nosema ceranae* and *Lotmaria passim* are two commonly encountered digestive tract parasites of the honey bee that have been associated with colony losses in Canada, the United States, and Europe. Though honey bees can be co-infected with these parasites, we still lack basic information regarding how they impact bee health at the individual and colony level. Using locally-isolated parasite strains, we investigated the effect of single and co-infections of these parasites on individual honey bee survival, and their responsiveness to sucrose. Results showed that a single *N. ceranae* infection is more virulent than both single *L. passim* infections and co-infections. Honey bees singly infected with *N. ceranae* reached < 50% survival eight days earlier than those inoculated with *L. passim* alone, and four days earlier than those inoculated with both parasites. Honey bees infected with either one, or both, parasites had increased responsiveness to sucrose compared to uninfected bees, which could correspond to higher levels of hunger and increased energetic stress. Together, these findings suggest that *N. ceranae* and *L. passim* pose threats to bee health, and that the beekeeping industry should monitor for both parasites in an effort correlate pathogen status with changes in colony-level productivity and survival.

## Introduction

The Western honey bee (*Apis mellifera* L.) is the world’s most intensively managed pollinator required for the pollination of many fruit, vegetable, and high-value cash crops. Unfortunately, the health of this pollinator is plagued by a number of factors, including the presence of a variety of pests and parasites^1^. The microsporidian *Nosema ceranae* and the trypanosomatid *Lotmaria passim* are two of these parasites. They are two globally-encountered digestive tract parasites of the honey bee^2–10^. *Nosema ceranae* was first described in 1996^11^, almost 90 years after its congener *N. apis*^12^, and has now largely replaced *N. apis* in most regions where both are present^13–20^. Similarly, the recently described *L. passim*^5^ has managed to outpace *Crithidia mellificae* (a trypanosomatid described from honey bees more than 50 years earlier)^21^ in terms of prevalence^5–9,22,23^.

Despite the cosmopolitan distribution of these two recently described digestive tract parasites^4,5,24^ that can co-occur^6,9,22^, we still lack a full understanding of the comparative effects of single and mixed *L. passim* and *N. ceranae* infections in honey bees. This is because *L. passim* is likely still underreported due to its emerging status, and it being previously misidentified as *C. mellificae*^5^. The organism has also largely been ignored by researchers due to it being considered benign^21^, despite it being reported as the most prevalent non-viral parasite in a cross-country study from the U.S.A.^24^. It is important to evaluate and understand comparative effects because different parasites and infections (single or mixed) can have varying effects on hosts, which could influence parasite management recommendations. For example, *Varroa destructor* and *Acarapis woodi*, two parasitic mites of the honey bee, have been shown to synergistically decrease honey bee colony survival when present as dual infestations, even though *A. woodi* is often considered to be inconsequential or variable in impact^25^. Due to the highly negative effect of this parasite when present with *V. destructor*, beekeepers were recommended to treat colonies for both parasites, in an era when the former organism was not routinely considered^25^.

In individual honey bees, *Nosema ceranae* infects the midgut epithelial cells^4,11^, and has the ability to degenerate midgut tissues^26,27^, alter foraging behaviour^28,29^, stimulate the immune system^30^, suppress the immune system^31,32^, alter learning and memory^33^, induce energetic stress^29,32^, and decrease nursing ability and lifespan of infected bees^28,34^. How *N. ceranae* affects honey bee colonies is much less clear, and varies with geographic location. In Spain, colonies infected with *N. ceranae* can experience decreases in colony size, honey production, brood-rearing capacity, and colony collapse^35–37^, but can also experience no pathological effects^38^. In western Europe, studies have shown no relationship between *N. ceranae* prevalence and colony mortality^39,40^. A study conducted in the United States showed that colonies with colony collapse disorder (CCD) had only slightly higher *N. ceranae* prevalence and abundance than control colonies^41^, while metagenomic analyses showed that infection with both *Nosema* spp. was a differentiating factor between healthy colonies and colonies with CCD^41,42^.

*Lotmaria passim* is found predominantly in the honey bee hindgut with a strong preference for the anterior rectum near the papillae and the distal portion of the ileum^5^. Though the preferred location of *L. passim* is known, the effects of the parasite on honey bee health are poorly understood. Within individual honey bees, there are conflicting reports regarding the parasite’s effect on longevity, as reports have shown both decreased^43–45^ or unaffected^46^ lifespans. At the colony level, *L. passim* has been correlated with increased winter colony mortality^6^, the collapse of colonies^9^, and can also be found concurrently with *N. ceranae*^6,9,22^.

*Nosema ceranae* has been shown to increase honey bee energetic stress leading to decreased survival^27^. We do not know how or if individual *L. passim* or mixed infections of the two parasites affect honey bee energetic stress, but a sucrose responsiveness assay could be used as a proxy, whereby increased responsiveness to sucrose could correspond to higher levels of hunger and increased energetic stress^29^. The sucrose responsiveness assay involves restrained bees and a series of sucrose solutions that vary in concentration^47^. The antennae of restrained bees are touched with droplets of these sucrose solutions in order of ascending concentration, and when a concentration of sucrose is acceptable, a bee responds by extending her proboscis^47,48^. Honey bees that respond to more concentrations of sucrose have increased responsiveness to sucrose (resulting in higher sucrose response scores [SRS]) compared to those responding to fewer concentrations^47,48^.

Given the ubiquitous reports of *N. ceranae* and/or *L. Passim* infections on honey bees, and the negative effects on bee health, an investigation is warranted to determine if novel management strategies are required for infections involving *L. passim*. Here, we investigate the effect of single and mixed *N. ceranae* and *L. passim* infections on individual honey bee survival and responsiveness to sucrose, using locally obtained parasite strains and honey bee stock. We hypothesize that parasitic infection will lead to increased sucrose responsiveness. We predict that honey bees inoculated with both parasites will have shorter lifespans and increased responsiveness to sucrose than those inoculated with either *N. ceranae* or *L. passim* due to the increased density and diversity of parasites, and the geographic separation of the parasites within the digestive tract, suggesting reduced interspecific competition.

## Results

### Survival

**Figure 1:**
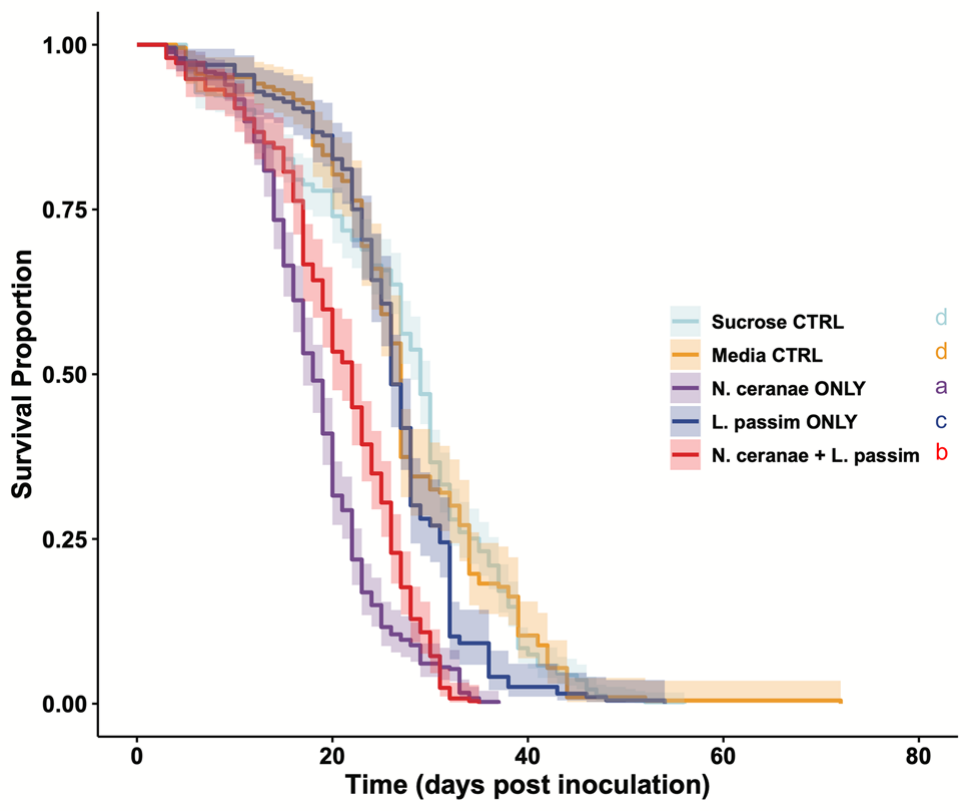
Survival of NEBs inoculated with one of five treatments modeled using a Mixed Effects Cox Model. Dark lines represent mean treatment survival across replicates, while the shading surrounding the dark lines represent 95% CI. Different letters represent significant differences between treatments (coxme; α = 0.05).

For inoculated newly-emerged bees (NEBs), there was an effect of treatment on survival (χ$$\begin{array}{c}2\\ 4\end{array}$$=353.2, *P* < *0.0001*, Fig. 1). NEBs inoculated with a parasitic treatment had significantly shorter lifespans than NEBs inoculated with sucrose or media control treatments. The *N. ceranae-*only treatment had the most negative effect on inoculated NEB survival, followed by the mixed-infection treatment, and then *L. passim-*only treatment. NEBs inoculated with *N. ceranae* only experienced < 50% survival four days earlier than NEBs inoculated with the mixed infection, eight days earlier than NEBs inoculated with *L. passim* only, and eight and 11 days earlier than NEBs inoculated with media and sucrose control groups respectively (Fig. 1). There was no difference in survival between sucrose and media control-inoculated NEBs (Fig. 1).

We confirmed the infection status of 60 NEBs across all 5 treatments and 4 replicates. All NEBs examined from the sucrose and media control treatments were free of both *N. ceranae* and *L. passim* across all replicates. All NEBs examined from the *N. ceranae* only, *L. passim* only, and mixed infection treatments were positive or negative for their respective treatments. No cross-contamination was observed.

### Sucrose responsiveness assay

**Figure 2:**
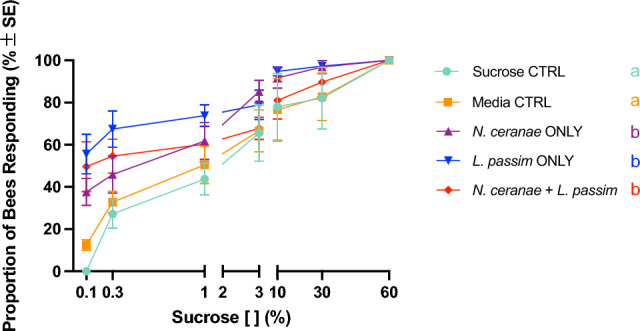
Proportion of NEBs inoculated with one of five treatments responding to a sucrose gradient at 16 days post inoculation (dpi). Each point represents the mean treatment response across four replicates ± SE, with different letters representing significant differences among treatments (glmer; α = 0.05).

**Figure 3:**
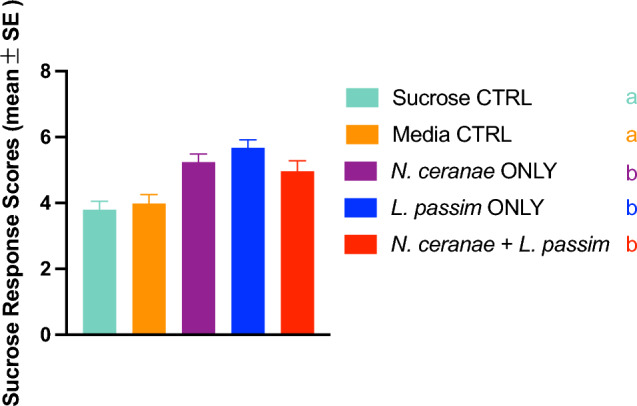
Sucrose responsiveness scores (SRS) for bees inoculated with one of five parasite treatments at 16 dpi. SRS were calculated by summing the number of sucrose concentrations in the series to which a bee responded by extending her proboscis. Each bar represents the mean treatment score across four replicates ± SE, with different letters representing significant differences among treatments (dunn.test; α = 0.05).

Overall, there was an effect of parasite treatment on honey bee responsiveness to sucrose (χ$$\begin{array}{c}2\\ 4\end{array}$$=39.686 *P* < *0.0001*, Fig. 2) as well as on the SRS of individual bees (χ$$\begin{array}{c}2\\ 4\end{array}$$=39.556 *P* < *0.05*, Fig. 3).

We confirmed the infection status and parasite density of 41 randomly chosen NEBs assayed for sucrose responsiveness at 16 dpi in 2019 and 2021 (4 NEBs per treatment per year, except for the *L. passim*-only treatment in 2021 where 5 NEBs were examined (see Supplemental Table 4 for densities). There was no difference in *N. ceranae* spore density between *N. ceranae* only and mixed infection NEBs in 2019 (*t* = *1.07,* df = 5.20*, P* = *0.33*) or 2021 (*t* = *0.77,* df = 3.20, *P* = *0.49*), and there was no difference in spore density between years for the *N. ceranae* only treatments (*t* = *2.00,* df = 4.64, *P* = *0.11*) or mixed infections (*t* = *1.09*, df = 3.30, *P* = *0.35*). There was no difference in *L. passim* cytb density between *L. passim* only and mixed infection NEBs in 2019 (*t* = *0.04,* df = 4.41, *P* = *0.97*) or 2021 (*t* = *2.09*, df = 3.00, *P* = *0.13*), and there was no difference in *L. passim* cytb density between years for the *L. passim* only treatments (*t* = *2.56*, df = 3, *P* = *0.08*) or mixed infections (*t* = *1.14*, df = 3.01, *P* = *0.34*) (see Table 1). All NEBs examined from the sucrose and media control treatments were negative for both *N. ceranae* and *L. passim*. All NEBs examined from the *N. ceranae* only, *L. passim* only, and mixed infection treatments were positive or negative for their respective treatments, and no cross-contamination was observed (Supplementary Table 4).

**Table 1:** Parasite densities for honey bees examined at the end of the sucrose responsiveness assay (16dpi).

Year	Parasite	Treatment	Mean density ± SE	n
2019	*N. ceranae*	*N. c*	7.05 × 10^7^ ± 8.43 × 10^6^	4
2019	*N. ceranae*	*N. c* + *L. p*	4.49 × 10^7^ ± 5.56 × 10^6^	4
2019	*L. passim*	*L. p*	2.12 × 10^6^ ± 8.29 × 10^5^	4
2019	*L. passim*	*N. c* + *L. p*	2.74 × 10^6^ ± 2.14 × 10^6^	4
2021	*N. ceranae*	*N. c*	8.98 × 10^7^ ± 4.60 × 10^6^	4
2021	*N. ceranae*	*N. c* + *L. p*	1.10 × 10^8^ ± 2.50 × 10^7^	4
2021	*L. passim*	*L. p*	1.87 × 10^3^ ± 5.69 × 10^2^	5
2021	*L. passim*	*N. c* + *L. p*	1.57 × 10^5^ ± 7.44 × 10^4^	4

After the sucrose responsiveness assay was complete, all responding NEBs were individually frozen at – 20 °C until processing occurred. At processing, 4 (or 5) NEBs were randomly chosen from each treatment and year to confirm infection status and density via microscopy (*N. ceranae*, spores/bee) and qPCR (*L. passim*, cytb copies/bee). No significant differences in parasite density exist between treatments or across years.

## Discussion

This study is the first to examine the effects of locally-obtained single and mixed *N. ceranae* and *L. passim* infections under controlled inoculation conditions on honey bee survival and energetic stress. Our survival curve which followed parasite-inoculated and uninoculated NEBs to total mortality showed that honey bee lifespan was negatively affected by both single and mixed parasitic infections. Though we did experience some total mortality of bees in specific cages early in this experiment, this was attributable to clogged sucrose feeders, rather than the effects of parasitization. Our sucrose responsiveness assay illustrated that inoculation with both single and mixed infections significantly increased honey bee responsiveness to sucrose, regardless of the infection type. This increased responsiveness was driven by the high SRS that parasitized NEBs had compared with control NEBs, which suggests that parasitized NEBs are experiencing higher levels of hunger due to increased energetic stress caused by the presence of parasites. These findings add to the body of literature which indicate *N. ceranae* is virulent in honey bees (especially to inoculated NEBs maintained in cages on liquid carbohydrates only), and the small but growing body of literature that suggests *L. passim* is pathogenic to honey bees on its own, but is not as virulent as *N. ceranae*^4,6,24,43–46,49–51^.

Currently, the literature is divided as to whether interactions between *N. ceranae* and *L. passim* occur due to their geographic separation within the honey bee digestive tract^22,46^. Tritschler et al.^22^ hypothesized no interaction occurs between *N. ceranae* and *L. passim* based on parasite quantities in field-collected honey bees, while Arismendi et al.^7^ suggested that synergism occurs between the parasites based on a honey bee survival curve experiment. In the current study, contrary to our prediction, NEBs inoculated with the mixed infection had longer lifespans than NEBs inoculated with *N. ceranae* only, and shorter lifespans than NEBs inoculated with *L. passim* only. This finding supports neither of the previously mentioned hypotheses, but instead supports the concept that immunomodulation is occurring (i.e. stimulation, or in this case, suppression of parts of the immune system), which is what Schwarz and Evans^28^ found in honey bees that had been inoculated with *N. ceranae* and *C. mellificae*. Although the previous authors did not monitor honey bee survival or parasite density, the patterns observed in their gene expression study mimic the patterns of NEB survival in our survival curve study having similar treatment groups. NEBs inoculated with a mixed infection in Schwarz and Evans^28^ mounted a more moderate response to infection (three antimicrobial peptides [AMPs] induced) than NEBs inoculated with *N. ceranae* only (five AMPs), and a more severe response than NEBs inoculated with *C. mellifcae* only (2 AMPs). Though we did not collect gene expression data, the phenomenon of increased survival and more moderate immune responses after inoculation with mixed infections has also been observed in other related host-parasite systems. In *Rhodnius prolixus*, bugs inoculated with both *Trypanosoma cruzi* and *T. rangeli* had increased survival, reproduction, and overall fitness compared to those inoculated with either *T. cruzi* or *T. rangeli* alone^52^. *Rhodnius prolixus* inoculated with both *T. cruzi* and *Beauvaria bassiana* exhibited increased survival compared to those inoculated with *T. cruzi* only^53^, while *Meccus pallidipennis* inoculated with both *T. cruzi* and *Metarhyzium anisopliae* had increased survival compared to those inoculated with either *T. cruzi* or *M. anisopliae* alone, and lower levels of phenyloxidase in hemolymph compared to those inoculated with only *T. cruzi*^54^. Garcia et al.^54^ and Peterson et al.^53^ suggested that *T. cruzi* exerts a protective effect against fungal infections as well as other trypanosomatid infections. The patterns of NEB survival observed in our survival curve study mimic the patterns of gene expression observed in Schwarz and Evans^28^, and collectively suggest immunomodulation is occurring, and that trypanosomatids may have a protective effect against *N. ceranae*. However, future experiments should endeavour to include gene expression data along with survival data to fully support this hypothesis.

Additionally, it is possible that synergism was not observed in our study as it was in Arismendi et al.^46^ due to differences in experimental design. Local *L. passim* and *N. ceranae* strains were used in both studies, meaning differences in results could, in part, be due to strain variation. In our study, we individually inoculated NEBs with *N. ceranae* and *L. passim* to ensure they received the desired density of both parasites (1.0 or 1.2 × 10^5^ respectively). Conversely, Arismendi et al.^46^ used NEBs obtained from colonies naturally infected with *L. passim* at a density of 1.0 ± 0.6 × 10^3^, and then individually inoculated NEBs with *N. ceranae* at a density of 1.0 ± 0.3 × 10^5^ as required. The differing densities of *L. passim* used in each experiment, as well as the differences in the order of parasite inoculation could have also contributed to variation in survival^53^. Differences in the diets that the NEBs were provisioned could also have influenced NEB survival. Honey bees infected with *N. ceranae* when provisioned on high quality pollen (protein) diets, exhibit increased survival despite an increased spore load, compared to those provisioned on low quality or no pollen diets^51,55,56^. The increased quantity and quality of pollen found in our pollen patties could be a factor contributing to the increased survival of NEBs inoculated with both *N. ceranae* and *L. passim* compared to those in Arismendi et al.^46^. One final reason Arismendi et al.^46^ may have observed a synergism that we did not is due to the difference in the length of the two experiments. The duration of the survival curve for Arismendi et al.^46^ was 20 days, whereas the survival curve in the current study ended with total mortality. It is possible that if Arismendi et al.^46^ increased the length of their experiment, that the results of the two studies would have been similar.

Though the results of the sucrose responsiveness assay did not completely reflect our prediction, the fact that parasite-inoculated NEBs had increased responsiveness to sucrose and higher SRS than control-inoculated NEBs is not surprising as parasites do possess the ability to modify the behaviour and physiology of their hosts^57,58^. *Nosema ceranae* has previously been shown to increase energetic stress (via sucrose responsiveness and molecular markers) and decrease the lifespan of honey bees^4,29^, both of which we observed in the current study. *Lotmaria passim* has also been shown to increase energetic stress (assessed via molecular markers) and may decrease the lifespan of bees^43–46^. Given *that L. passim* appears to be less virulent than *N. ceranae*, it was surprising to see NEBs inoculated with the *L. passim-*only treatment having (numerically) higher SRS than NEBs inoculated with *N. ceranae* only, despite having a longer lifespan. This finding may correspond to *L. passim*-infected bees having higher levels of hunger, and in turn, increased energetic stress. Taken together with the longer lifespan, this suggests that honey bees infected with only *L. passim* may be able to better compensate for the long-term negative effects of infection (e.g., decreased lifespan) simply by consuming greater quantities of resources when they are present, which could be quantified in future experiments. This type of compensation has been observed several times in Hymenoptera under various starvation and infection scenarios^51,59–62^. The (numerically) lower SRS of bees infected with *N. ceranae* either alone or in the mixed infection indicate that if these bees are compensating for the negative effects of infection via diet consumption, that the quality of resources may also play a role in the bees’ ability to compensate when *N. ceranae* is present. The influence of protein (pollen) quality in *N. ceranae* infections has been observed previously^51,64^. Bees that were infected with *N. ceranae* that were provisioned on the highest quality protein diet consumed more diet than *N. ceranae*-infected bees provisioned on lower quality diets^51,64^. In addition to being a highly virulent parasite, *N. ceranae* is also dependent on its host’s nutritional status for development because it is amitochondriate^4^. Therefore, having a bee receive and provide better quality nutrition should be to the benefit of *N. ceranae* (and the bee), and may be why we observed (numerically) lower SRS in bees infected with *N. ceranae*.

We observed no significant difference in parasite density across treatments or years for honey bees at the end of the sucrose responsiveness assay (16 dpi). This finding, along with the increased lifespan for NEBs inoculated with *L. passim* alone, supports the previously mentioned trade-off, where the presence of *L. passim* seems to allow honey bees to compensate for the negative effects of infection via increased food consumption. Increased sucrose consumption has been both suggested and observed for honey bees infected with other parasites such as *N. ceranae*^29,63^. Additionally, honey bees infected with *N. ceranae* that had access to high quality pollen diets as adults had increased survival^51,56,59,64^ and increased spore loads compared to those that did not^51,55,59^. For bees inoculated with *N. ceranae* either alone or with the mixed infection in our experiment, the fact that no differences in parasite densities across treatments were seen, coupled with decreased lifespans, again suggest that resource quality may also play a role in the ability of bees to compensate for infection. Though we have neither sucrose nor pollen consumption data for the current experiment, and we did not manipulate diet quality, it is reasonable to assume that honey bees with higher SRS (parasitized bees) would also be consuming more sucrose, and perhaps pollen as young adults, potentially allowing them to effectively immunomodulate and thereby increase survival^56,65^. To determine if increased food consumption and/or diet quality leads to infected honey bees having longer lifespans via immunomodulation, one could manipulate diet quality, and monitor consumption as well as immune gene expression and survival over time. Given that *N. ceranae* is an intracellular, amitochondriate parasite that depends on the nutritional status of the honey bee for development^4^, we would expect to see increased consumption of higher quality diet (sucrose and pollen), and better immunomodulation by the bees consuming high quality diet. Since *L. passim* is an extracellular parasite that may use glucose as a source of energy^66^, we would expect to see consumption, particularly of sucrose, increase with decreasing quality, and immunomodulation to be stable across diet treatments as long as bees could vary their consumption accordingly.

Initially, we were surprised at the disparity between *N. ceranae* and *L. passim* densities within the NEBs examined from the sucrose responsiveness assay because similar dosages and the same inoculation technique were used. However, given that *N. ceranae* is an intracellular parasite of the honey bee midgut, and *L. passim* is an extracellular parasite of the honey bee hindgut, differences in density could be related to differences in reproductive strategies, and the length of time required for the parasites to complete a reproductive cycle. In a lepidopteran cell line, *N. ceranae* is able to complete its life cycle in 96 h^67^. Though we do not know the length of time *L. passim* requires to complete its life cycle within the honey bee digestive tract, or in culture media, we do know that *L. passim* cell densities in culture media can range from more than 20 × less to 33 × more than the initial inoculum density at 96 h after inoculation depending on the culture media used^44^. Furthermore, though both parasites could be transmitted via a fecal–oral route^4,5,43^ it is much more likely that a *L. passim* infection could be lost or reduced via a defecation event compared to a *N. ceranae* infection, due to its presence in the hindgut, leading to lower parasite density.

This study has illustrated that under standardized cage conditions, single and mixed *N. ceranae* and *L. passim* infections negatively affect honey bee survival, and their responsiveness to sucrose. These results confirm that *N. ceranae* is a highly virulent honey bee parasite^4,28–31,33,34^, and support what is currently known about *L. passim*, which is that the parasite is pathogenic to honey bees, but less virulent than *N. ceranae*^43,45,46^. Further studies are required to determine if the negative effects of the parasites observed in this study remain under differing experimental conditions such as parasite inoculation order, cell culture passage number, and parasite strain variation. Buendía-Abad et al.^43^ found that long-term in vitro* L. passim* cultures obtained from culture collections had reduced virulence compared to locally-obtained strains, because of increased cell culture passages. Benefits of using parasite strains obtained locally are twofold: (1) cell culture passage numbers are known and (2) virulence in local host populations can be determined. Additionally, further work should be done to determine if the negative effects of the parasites observed at the cage and individual-level in this study translate to the colony-level, which would warrant the development of novel parasite management strategies. Because honey bee colonies have a strong buffering capacity^68^, it is possible that the effects observed in the current study may not translate into the field. However, it is also possible that the cage-level effects would translate, as we have recently seen with *N. ceranae* and its effect on honey bee mortality^28,69^. Decreased lifespans, and increased responsiveness to sucrose could manifest as precocious foraging, and smaller, less-productive populations at the colony-level, which we have seen before with *N. ceranae*^4^. If precocious foraging, and smaller populations, are observed particularly for colonies infected with *L. passim* or *L. passim* and *N. ceranae*, novel management strategies, such as those involving the application of phytochemicals should be further explored^70^.

## Conclusion

Our study, for the first time, illustrates the negative effects of single *L. passim* and mixed *L. passim* and *N. ceranae* infections on honey bee survival and sucrose responsiveness under controlled inoculation conditions with local parasite strains. Based on the results of this study, we recommend that beekeepers continue to monitor their colonies for *N. ceranae*, and begin to routinely monitor for *L. passim* in an effort to improve honey bee health by correlating parasite diagnosis with colony-level changes that could affect survival and productivity.

## Methods and materials

### Parasites

An axenic culture of *L. passim* isolated from the dissected ileum of an adult honey bee worker at Agriculture and Agri-Food Canada’s (AAFC) Beaverlodge Research Farm (55° 11′ 43.0″ N; 119° 17′ 57.3″ W) was established in the fall of 2016 (Supplementary Table 1). The culture was grown in a water-jacketed incubator at 25 ± 0.1 °C (model 3326, Forma Scientific, Ottawa, ON, Canada)^5^ to high density in Schneider’s *Drosophila* medium (Cat# 21720024, Fisher Scientific, Ottawa, ON, Canada), supplemented with 10% fetal bovine serum (Cat# 16140071, Fisher Scientific, Ottawa, ON, Canada), and 100 IU/mL Penicillin-100 μg/mL Streptomycin-2.5 μg/mL Amphotericin B (Cat# 30004CI, Fisher Scientific, Ottawa, ON, Canada). The culture was then cryopreserved in liquid nitrogen, and when needed, thawed, and grown to high density in 15 and 50 mL centrifuge tubes at 25 ± 0.1 °C in the supplemented Schneider’s *Drosophila* medium mentioned above. Prior to inoculation, *L. passim* cultures were centrifuged at 200×*g* for 10 min. After this initial centrifugation step, the supernatant was removed, and filtered through a 0.22 μm filter, while the pellet was resuspended in 1 mL of 1 × phosphate-buffered saline (PBS). The filtered supernatant was centrifuged once at 200×*g* for 10 min, the resulting supernatant removed, and any remaining pellet resuspended to 1 mL with a 1:10 sucrose (50% *w/v*): PBS (1 ×) solution. The resuspended *L. passim* pellet was successively washed and centrifuged twice in 1 mL of 1 × PBS at 200×*g* for 10 min. After a final resuspension of the pellet in 1 mL of 1 × PBS, a count was performed using a Helber Z30000 counting chamber (Cat# Z30000, Hawksley, Sussex, United Kingdom) to estimate the number of motile, flagellated *L*. *passim* cells/mL of culture.

*Nosema ceranae* spores were obtained from the dissected midguts of *N. ceranae*-infected adult *A. mellifera* workers at AAFC’s Beaverlodge Research Farm; the procedure for spore collection was adapted from MacInnis et al.^71^. After dissection, midguts were manually macerated in 1 mL of 1 × PBS in a Stomacher® 80 Biomaster Standard Bag (Cat# BA6040, Seward, West Sussex, United Kingdom) for 1 min, before maceration in a Stomacher® 80 blender (Cat # 030010019, Seward, West Sussex, United Kingdom) for 5 min. The macerate was then passed through a 40 μm cell strainer (Cat# 352340, Fisher Scientific) and rinsed with 15 ml of 1 × PBS. The resulting filtrate was then vacuum-filtered through a 10 μm separator (Cat# 60344, Pall Corporation, Ann Arbor, MI, USA) and rinsed with another 15 mL of 1 × PBS. The resulting 30 mL filtrate was then centrifuged at 800×*g* for 10 min, and the pellet resuspended in 1 mL of 1 × PBS. The 1 mL of *N. ceranae* spores in 1 × PBS was then treated with 100 IU/mL Penicillin-100 μg/mL Streptomycin (Cat# 15140122, Fisher Scientific) for 1 h to kill any contaminating bacteria^30^. The *N. ceranae* spores were then washed 3 times in 1 mL of 1 × PBS followed by centrifugation at 800×*g* for 10 min. After the final resuspension in 1 mL of 1 × PBS, a count was performed using a Helber Z30000 counting chamber to estimate the number of spores/mL^72^. *Nosema* spp. were verified via conventional polymerase chain reaction (PCR) techniques outlined in van den Heever et al.^73^ with the following modifications: 200 μL of macerate were used for DNA extractions; 75 ng of total DNA was amplified via PCR; and primers NoscRNAPol-F2/NoscRNAPol-R2 NosaRNAPol-F2/NosaRNAPol-R2 as well as thermal cycler settings in Gisder and Genersch^74^ were used to differentiate between *N. apis* and *N. ceranae*.

### Experimental bees

Frames of eclosing worker bees were collected from nonexperimental colonies managed by the Apiculture Program at AAFC Beaverlodge. Four to six frames from four to six different colonies confirmed to be *Nosema* spp.-free, and trypanosomatid-free via PCR were maintained in a 33 °C ± 1.0 °C programmable incubator (models I36NLC8, I36NLC9, Percival Scientific, Perry, IA) at any given time. Bees were collected from these frames daily, so that all newly-emerged bees (NEBs) used for experiments were < 24 h old, and free of any *Nosema* spp. and trypanosomatid spp. infections.

### Survival

Individual NEBs were orally-inoculated with 5 μL of one of the five following treatment groups diluted in 1:10 sucrose (50% *w/v*): PBS (1 ×) solution via a 10 μL pipette: sucrose control (1:10 sucrose:PBS solution only), media control (any resulting pellet from the centrifuged *L. passim* supernatant), *N. ceranae* only (1.0 × 10^5^ *N. ceranae* spores), *L. passim* only (1.2 × 10^5^ motile, flagellated *L. passim* cells), and *N. ceranae* + *L. passim* (1.0 × 10^5^ *N. ceranae* spores + 1.2 × 10^5^ motile, flagellated *L. passim* cells). After inoculation, NEBs were maintained individually in 15 mL centrifuge tubes for 30 min to ensure the inoculum was ingested (no inoculum droplets observed within the centrifuge tubes) before caging occurred. After this 30 min, NEBs that had not fully consumed their inoculum were discarded, while those that did were caged by treatment. Each of the five treatments consisted of two cages (A and B); each containing an average of 51 ± 0.6 inoculated NEBs, along with an average of 48 ± 0.7 uninoculated NEBs (thoraxes paint-marked to identify them) to provide social interaction, and to act as controls that received minimal handling (see Supplementary Table 2 for details). Cages (plastic cages used to hold NEBs) were maintained at 33 °C ± 1.0 °C in programmable incubators, and NEBs were provisioned on 50% *(w/v)* sucrose in a gravity feeder, and pollen patty in a diet tray *ad lib.* The pollen patty was prepared by Global Patties (Airdrie, Alberta, Canada) according to their standard recipe, but modified to include 25% [by weight] irradiated Canadian-collected *B. napus* pollen. The modified *(w/w)* recipe contained 46% sucrose syrup, 15% distillers dried yeast, 14% defatted soy flour, and 25% irradiated *B. napus* pollen. Diet was replaced every 72 h, and mortality was monitored daily until total mortality occurred for each treatment during the summer of 2018 when all four replicates were conducted. Dead bees were removed from cages as they appeared, and stored at − 20 °C for further processing. Unfortunately, some total cage mortality occurred that was not due to experimental infection (e.g. clogged sucrose feeders), but each treatment was accounted for in at least three of the four replicates (see Supplementary Table 2).

### Confirmation of infection

In order to confirm infection, and to ensure that cross-contamination did not occur between treatments, we examined NEBs that were dead at 15 dpi. If no NEBs were dead at 15 dpi, we took dead NEBs at the next time point they occurred (e.g. 17 dpi). We examined 2–4 dead NEBs per treatment group per replicate. We confirmed *N. ceranae* was the only *Nosema* spp. present in our experiment, and only present in treatments that contained *N. ceranae* via endpoint PCR as above (see *Nosema ceranae*) with the following modifications: the 25μL endpoint PCR reaction was comprised of 12.5 μL Accustart II PCR Supermix (Cat# 95137–500, VWR, Mississauga, ON, Canada); 0.5 μL of each forward and reverse primer was used (final concentration 0.2 μM per primer, 2.0 μL final volume); and 75 ng of DNA and nuclease-free water was included. We confirmed *L. passim* infections occurred only in treatments that were inoculated with *L. passim* using quantitative (q) PCR to detect copies of the *L. passim* cytochrome b (cytb) gene in each NEB. The qPCR reactions consisted of SSoAdvanced™ Universal SYBR® Green Supermix (BioRad Laboratories, Hercules, USA), genomic DNA, nuclease-free water, and LpCytb_F2, and LpCytb_R primers^75^ with RpS5 as a reference gene^74^. Amplification assays were performed in triplicate in a CFX384 Touch™ Real-Time Detection System (BioRad Laboratories, Hercules, USA. Thermal cycler settings were 3 min at 98 °C for initial denaturation/enzyme activation followed by 40 cycles of 10 s at 98 °C and 20 s at 60 °C. Specificity was checked by performing a melt-curve analysis from 65 to 95 °C in increments of 0.5 °C at 2 s per step.

### Sucrose responsiveness assay

Individual NEBs were orally-inoculated as above (see Survival). NEBs that did not readily consume their inoculum were discarded, while those that did were caged according to treatment. Each treatment consisted of one cage per replicate (total of 4 replicates per treatment). In 2019, cages consisted of 30 bees per treatment, while in 2020 and 2021, cages consisted of 40 bees per treatment. Cages were maintained and provisioned as above, with dead bees being removed from cages as they appeared. At 16 dpi, the inoculated NEBs were prepared for the sucrose responsiveness assay.

At 16 dpi inoculated NEBs were starved in their cages for 60 min prior to being collected and briefly cold anesthetized on ice until immobile^48^. Each inoculated NEB was then restrained in a harness (a cut-off portion of a drinking straw) using a thin piece of parafilm placed between the head and the thorax (see Supplementary Fig. 1 and Scheiner et al.^76^ for an additional example). Special care was taken to ensure that the inoculated NEBs could still freely move their proboscises and antennae after restraint. These restrained NEBs were then starved for an additional 4.5–5 h before the sucrose responsiveness assay began. The antennae of restrained NEBs were presented with a concentration series of 0.1, 0.3, 1, 3, 10, and 30% sucrose^47^ with 60% as a positive control. NEBs were assayed in ascending order of sucrose concentration to decrease potential sensitization that can occur with higher sucrose concentrations. After each sucrose presentation, water was provided to the antennae to control for sensitization or habituation^47,48^. The interstimulus interval (interval between successive sucrose concentrations) varied between 1–2 min depending on the number of individuals being assayed at any one time, usually between 10 and 25. A NEB was observed to ‘respond’ by fully extending its proboscis when a drop of sucrose was touched to its antennae. Small movements of a proboscis that did not result in full extension were not considered responsive. NEBs that responded to water, responded inconsistently, or failed to respond to 60% sucrose were excluded from further analyses. After the sucrose responsiveness assay was complete, all NEBs that responded to the assay were frozen at − 20 °C for further processing.

### Confirmation and quantification of infection

We confirmed infection, quantified parasite load, and ensured cross-contamination did not occur in 4 randomly chosen NEBs per treatment that were assayed for sucrose responsiveness in 2019 and 2021. We confirmed *N. ceranae* was the only *Nosema* spp. present, and only present in treatments that contained *N. ceranae*-inoculated bees as above. We also quantified the number of *N. ceranae* spores/mL using a Helber Z3000 counting chamber as above (see *Nosema* ceranae), and detected copies of the *L. passim* cytb gene/NEB using the same technique as above (Confirmation of infection-(survival experiment)), but then also quantified the number of copies of the *L. passim* cytb gene/NEB via absolute quantification using the standard curve method. Standard curves were prepared from plasmids harbouring the target amplicons with copy numbers diluted from 10^8^ to 10^2^ (see Supplemental Table 3).

### Statistical analyses

Statistical analyses were performed in ‘R’studio v.4.2.1 for Mac OS X^77^. Survival curve data were analyzed using a Mixed Effects Cox Model (coxme, 2.2–18.1, coxme) with treatment as the predictor variable, and ‘cage’ nested with ‘replicate’ as a random effect. This was then followed by Anova (Anova, 3.1–0, car) to determine if there was an effect of treatment on survival. Post-hoc tests were then completed using emmeans (emmeans, 1.8.5, emmeans) with a Benjamini Hochberg correction for multiple comparisons to differentiate between treatment effects. Sucrose responsiveness data were analyzed using a generalized linear mixed effects model with a binomial distribution. Response to sucrose was used as the response variable, parasite treatment as the predictor variable, and ‘bee’ as a random effect. The significance of the predictor variable was evaluated using an *F*-test (Anova, 3.1–0, car), and multiple comparisons were performed (glht, 1.4–20, multcomp). Model fit was assessed by plotting the scaled residuals, examining Levene’s test for homogeneity of variance, and Kolmogorov–Smirnov test for overdispersion (simulateResiduals, 0.4.6, DHARMa). SRS were calculated by summing the number of sucrose concentrations in the series to which a bee responded by extending her proboscis^78^. SRS were then evaluated using a Kruskall-Wallis rank sum test followed by Dunn’s test of multiple comparisons (Dunn.test, 1.3.5, dunn.test) to determine if treatment had an effect on SRS. We also compared parasite density between single and mixed infection treatment groups within and between years for bees from the sucrose responsiveness assay using Welch’s *t* tests.

### Supplementary Information

Supplementary Information 1.

Supplementary Information 2.

Supplementary Information 3.

## Data Availability

All data generated or analyzed during this study are included in the published article and its supplementary information files.
