# Supplementary material for: A tale of two parasites: Responses of honey bees infected with Nosema ceranae and Lotmaria passim
Source: Sci Rep. 2023 Dec 18;13:22515. doi: 10.1038/s41598-023-49189-9 (PMC10728187; doi:10.1038/s41598-023-49189-9)
Supplement: Supplementary file 3 — Supplementary Information 3. [file 41598_2023_49189_MOESM3_ESM.docx]

**SUPPLEMENTARY INFORMATION**

**A tale of two parasites: Responses of honey bees infected with *Nosema ceranae* and *Lotmaria passim***

Courtney I. MACINNIS, Lien T. LUONG, Stephen F. PERNAL

Page 2 Supplemental Figure 1. Newly-emerged bees (NEBs) prepared in harnesses for sucrose responsiveness assay (SRS)

Page 3 Supplemental Table 1. Amplified Cytochrome b product sequenced for trypanosomatid species identification

Page 4 Supplemental Table 2. Number of (NEBs)/cage/treatment/replicate of the survival curve experiment

Page 5 Supplemental Table 3. qPCR primers and standard curve information for RpS5 and *Lotmaria passim* cytb

Page 6-7 Supplemental Table 4. Confirmation of infections and parasite densities in NEBs from the sucrose responsiveness assay

Raw data for the survival curve analyses is available in the separate file “surv.xlsx” in the Supplementary Information

Raw data for the sucrose responsiveness assay analyses is available in the separate file “srs.xlsx” in the Supplementary Information

Supplemental Figure 1. NEBs prepared in harnesses (cut-off portions of drinking straws and parafilm) for the sucrose responsiveness assay


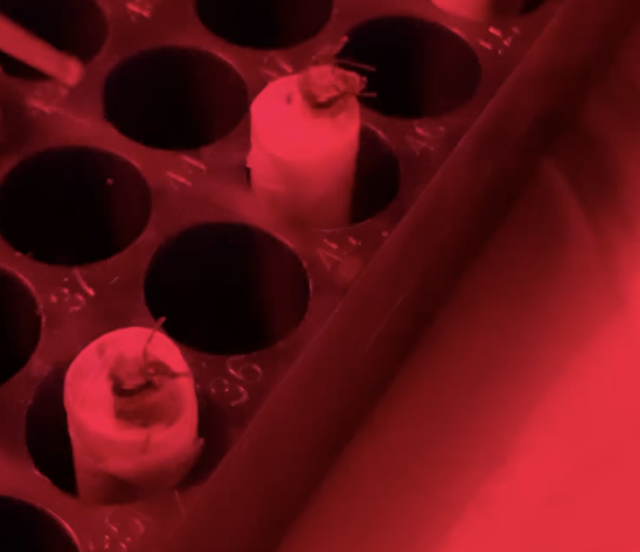


Supplemental Table 1. Amplified Cytochrome b product sequenced for species identification

| **Organism** | **Target** | **Product sequence** | **Identity (%)** | **Accession number** |
| --- | --- | --- | --- | --- |
| *L. passim* | Cytochrome b | TCGTGTAAAGCGGAGAAAGAAGAAAAGGCTTTTAACGT  CAGGTTGCTTATTAAGAGTATATGGAGTAGGTTTTAGTT  TAGGTTTTTTTATATGCATGCAAATTATATGTGGTGTATG  TTTAGCATGATTATTTTTTAGCTGTTTTATATGTACTAATT  GATATTTTGTTTTATTTTTATGAGATTTTGATTTAGGTTTT  GTAATACGAAGTGCACATATATGCTTTACATCATTATTAT  TCTTTTTACTGTATGTTCATATATTTAAAGCGATCGTTTTA  ATAATTTTATTTGATACTCATATTTTAGTATGAGCAGTAG  GTTTTATCATATATATATTCATAGTAGTTATAGGTTTTATT  GGATATGTATTACCATGTACAATGATGTCTTATTGAGGTC  TAACTGTTTTTAGTAATATTTTAGCAACAGTACCAGTTATT  GGTGTTTGGCTATGTTATTGAATATGAGGTAGTGAGTTTA  TAAATGATTTTACACTATTAAAATTACATGTGCTACATGTA  TTATTGCCATTTGTTTTAATATTAGTTATAGTTATGCACTT  ATTTTGCTTACATTATTTTATGAGCTCGGATGGTTTTTGT  GATCGTTTTGCTTTTTATTGTGAACGTTTGTGT | 98.69 | KM980180.1 |

| Supplemental Table 2. Number of NEBs/cage/treatment/replicate in the survival curve experiment | | | | | | |
| --- | --- | --- | --- | --- | --- | --- |
| **Replicate** | **Treatment** | **Cage A INOC** | **Cage A UNINOC** | **Cage B INOC** | **Cage B UNINOC** |  |
| 1 | Control | 50 | 54 | 51 | 49 |  |
| 1 | Media control | 52 | 56 | - | - |  |
| 1 | *N. ceranae* only | 55 | 43 | 52 | 49 |  |
| 1 | *L. passim* only | - | - | 50 | 48 |  |
| 1 | *N. ceranae* + *L. passim* | 52 | 50 | 44 | 47 |  |
| 2 | Control | 51 | 45 | 54 | 42 |  |
| 2 | Media control | - | - | - | - |  |
| 2 | *N. ceranae* only | 54 | 44 | 50 | 48 |  |
| 2 | *L. passim* only | 44 | 48 | - | - |  |
| 2 | *N. ceranae* + *L. passim* | 52 | 52 | - | - |  |
| 3 | Control | 54 | 44 | 53 | 51 |  |
| 3 | Media control | 49 | 46 | 54 | 46 |  |
| 3 | *N. ceranae* only | - | - | 49 | 50 |  |
| 3 | *L. passim* only | - | - | 52 | 57 |  |
| 3 | *N. ceranae* + *L. passim* | - | - | - | - |  |
| 4 | Control | 51 | 48 | 51 | 47 |  |
| 4 | Media control | - | - | 48 | 49 |  |
| 4 | *N. ceranae* only | 47 | 47 | 54 | 47 |  |
| 4 | *L. passim* only | 50 | 46 | - | - |  |
| 4 | *N. ceranae* + *L. passim* | 53 | 49 | 48 | 48 |  |
|  |  |  |  |  |  |  |
| INOC= inoculated NEBs | |  |  |  |  |  |
| UNINOC = Uninoculated NEBs for social interaction | | |  |  |  |  |
| -= Not monitored due to mortality that occurred which was not due to experimental infection | | | | | |  |

| Supplemental Table 3. qPCR primers, and standard curve information used to determine *Lotmaria passim* infection status in survival curve and sucrose  responsiveness NEBs as well as *L. passim* loads in sucrose responsiveness NEBs in this study. |
| --- |

| **Assay** | **Primer** | **Sequence** | **NT** | **Tm**  **(°C)** | **Size** | **Melt temp**  **(°C)** | **R^2^** | **Efficiency**  **(%)** | **Slope** | ***Y*-int** | **Reference** |
| --- | --- | --- | --- | --- | --- | --- | --- | --- | --- | --- | --- |
| RpS5 qPCR | RpS5 F | AATTATTTGGTCGCTGGAATTG | 22 | 51.6 | 115 | 77.5 | 0.998 | 96.5 | -3.409 | 38.857 | Evans *et al.* 2006 |
|  | RpS5 R | TAACGTCCAGCAGAATGTGGTA | 22 | 55.9 |  |  |  |  |  |  |  |
| *L. passim*  cytb qPCR | Lpcytb_F2 | AGTATGAGCAGTAGGTTTTATTATA | 25 | 49.3 | 146 | 75.5 | 0.994 | 86 | -3.699 | 37.973 | Vejnovic *et al*. 2018 |
|  | Lpcytb_R | GCCAAACACCAATAACTGGTACT | 23 | 55.4 |  |  |  |  |  |  |  |

| Supplemental Table 4. Confirmation of infection and parasite densities in NEBs from the sucrose responsiveness assay. After the sucrose responsiveness assay was complete, all responding NEBs were individually frozen in 1.5mL microfuge tubes and stored at -20°C until processing occurred. At processing, 4 (or 5) NEBs from each treatment in both 2019 and 2021 were randomly chosen to confirm infection status and parasite density via microscopy (*N. ceranae*) and qPCR (*L. passim*) | | | | | | | |
| --- | --- | --- | --- | --- | --- | --- | --- |
| **Year** | **Trt** | **ID#** | ***N.c*** | ***N.a*** | ***L.p*** | ***N. ceranae* spores/bee** | **cytb copies/bee NORM** |
| 2019 | CTRL | 1 | - | - | - | 0 | 0 |
| 2019 | CTRL | 2 | - | - | - | 0 | 0 |
| 2019 | CTRL | 3 | - | - | - | 0 | 0 |
| 2019 | CTRL | 4 | - | - | - | 0 | 0 |
| 2019 | MEDIA | 5 | - | - | - | 0 | 0 |
| 2019 | MEDIA | 6 | - | - | - | 0 | 0 |
| 2019 | MEDIA | 7 | - | - | - | 0 | 0 |
| 2019 | MEDIA | 8 | - | - | - | 0 | 0 |
| 2019 | NC | 9 | + | - | - | 46500000 | 0 |
| 2019 | NC | 10 | + | - | - | 76500000 | 0 |
| 2019 | NC | 11 | + | - | - | 85750000 | 0 |
| 2019 | NC | 12 | + | - | - | 73500000 | 0 |
| 2019 | LP | 13 | - | - | + | 0 | 1643336.103 |
| 2019 | LP | 14 | - | - | + | 0 | 3664332.509 |
| 2019 | LP | 15 | - | - | + | 0 | 2472.551 |
| 2019 | LP | 16 | - | - | + | 0 | 3189343.126 |
| 2019 | LPNC | 17 | + | - | + | 80250000 | 203398.569 |
| 2019 | LPNC | 18 | + | - | + | 77750000 | 6993867.836 |
| 2019 | LPNC | 19 | + | - | - | 70750000 | 0 |
| 2019 | LPNC | 20 | + | - | + | 97000000 | 1033041.891 |
| 2021 | CTRL | 21 | - | - | - | 0 | 0 |
| 2021 | CTRL | 22 | - | - | - | 0 | 0 |
| 2021 | CTRL | 23 | - | - | - | 0 | 0 |
| 2021 | CTRL | 24 | - | - | - | 0 | 0 |
| 2021 | MEDIA | 25 | - | - | - | 0 | 0 |
| 2021 | MEDIA | 26 | - | - | - | 0 | 0 |
| 2021 | MEDIA | 27 | - | - | - | 0 | 0 |
| 2021 | MEDIA | 28 | - | - | - | 0 | 0 |
| 2021 | NC | 29 | + | - | - | 89000000 | 0 |
| 2021 | NC | 30 | + | - | - | 100250000 | 0 |
| 2021 | NC | 31 | + | - | - | 78000000 | 0 |
| 2021 | NC | 32 | + | - | - | 92000000 | 0 |
| 2021 | LP | 33 | - | - | + | 0 | 653.337 |
| 2021 | LP | 34 | - | - | + | 0 | 1332.646 |
| 2021 | LP | 35 | - | - | - | 0 | 0 |
| 2021 | LP | 36 | - | - | + | 0 | 2183.409 |
| 2021 | LPNC | 37 | + | - | + | 73750000 | 21299.723 |
| 2021 | LPNC | 38 | + | - | + | 183250000 | 83279.668 |
| 2021 | LPNC | 39 | + | - | + | 86250000 | 363157.514 |
| 2021 | LPNC | 40 | + | - | + | 94750000 | 159154.591 |
| 2021 | LP | 41 | - | - | + | 0 | 3292.705 |
| Trt=Treatment | |  |  |  |  |  |  |
| ID#=Bee ID | |  |  |  |  |  |  |
| *N.c*= *N.ceranae* | |  |  |  |  |  |  |
| *N.a*= *N.apis* | |  |  |  |  |  |  |
| *L.p*= *L.passim* | |  |  |  |  |  |  |
| cytb copies/bee NORM = *L. passim* cytb copies/bee normalized to RpS5 | | | | | | |  |
